# *Echinococcus granulosus* Prevalence in Dogs in Southwest Nigeria

**DOI:** 10.1155/2014/124358

**Published:** 2014-05-12

**Authors:** Oyeduntan Adejoju Adediran, Temitope Ubaidat Kolapo, Emmanuel Chibuike Uwalaka

**Affiliations:** Department of Veterinary Microbiology and Parasitology, Faculty of Veterinary Medicine, University of Ibadan, Ibadan, Nigeria

## Abstract

Echinococcosis is a public health parasitic disease that is cosmopolitan (*Echinococcus granulosus*) in its distribution. Domestic dogs (*Canis familiaris*) have been recognised as the definitive host of the parasite. The present study was carried out to determine the prevalence of canine echinococcosis in Southwest Nigeria using direct enzyme linked immunosorbent assay (ELISA) to detect sera antigen. Two hundred and seventy-three (273) canine sera were tested for the presence of *Echinococcus* antigen. Purpose of keeping (hunting or companion), age (young or adult), and sex of each dog were considered during sampling. Total prevalence recorded was 12.45% (34/273). There was significant difference (*P* < 0.05) between hunting (15.94%) and companion dogs (1.52%) but there was no significant difference (*P* > 0.05) between young and adult dogs. There was no association between sex and prevalence of canine echinococcosis. The result of this study established the presence of canine echinococcosis in Southwest Nigeria; thus there is the possibility of occurrence of zoonotic form of the disease (human cystic hydatid diseases) in the region.

## 1. Introduction

Echinococcosis is a zoonotic parasitic tapeworm infection caused by the larval stage of several species belonging to the genus* Echinococcus*. There are four main species of* Echinococcus* affecting man and animals, and they include* Echinococcus granulosus*,* E. multilocularis*,* E. oligarthrus*, and* E. vogeli*. Echinococcosis has been termed an emerging/reemerging disease [1, 2]. The life cycle of the tapeworm (*Echinococcus granulosus*) is sustained between the definitive hosts, which are dogs and exhibit canine echinococcosis, and herbivores, the intermediate host in which cystic hydatid disease occurs. Echinococcosis has been identified as a zoonosis in rural livestock-raising areas where humans cohabit with dogs fed on raw livestock offal [3]. Feeding dogs with raw viscera of infected animals contributes to perpetuating this cycle [4]. Humans get infected by accidental ingestion of eggs from tapeworm-infected dogs and develop cystic lesions, principally in liver and lungs, after several years [5]. It also results in significant economic loss to the meat industry through condemnation of infected organs in food animals [6].

*E. granulosus* and* E. multilocularis* are species of major public health importance and are responsible for virtually all the human and animal burden of the disease causing human cystic echinococcosis (CE) and alveolar echinococcosis (AE), respectively [7].* E. granulosus* has a worldwide geographic distribution and occurs in all continents. High parasite prevalence is found in parts of Eurasia, Africa, Australia, and South America [8]. Cystic echinococcosis is regarded as a global public health concern and is endemic in many parts of the world [9] including sub-Saharan Africa [10], which Nigeria is a part of. The first study on canine echinococcosis in Nigeria carried out in Bauchi Plateau zone in the northern part of the country [11] reported no prevalence although low numbers of dogs were examined. Since then very few studies have been carried out on the prevalence of the parasite. Further, Nigeria is a country that has witnessed tremendous increase in livestock production and dog keeping for hunting and as pets in the rural areas and as guards in the urban areas. Because of its public health significance, human hydatid disease has been the subject of considerable research throughout the world and is considered by The World Health Organization (WHO) as one of the most widespread parasitic diseases and also one of the most costly to be treated and prevented in terms of public health [8]. Although regarded as widespread, there have been few recent studies on hydatidosis in farm animals [12–15] and no study on canine echinococcosis in the last two decades in Nigeria, hence the need for the study.

This study therefore was carried out to determine the prevalence of* Echinococcus granulosus* in dogs and management practices that may predispose to the perpetuation of the infection in dogs in Southwest Nigeria.

## 2. Materials and Methods

### 2.1. Study Area

**Figure 1:**
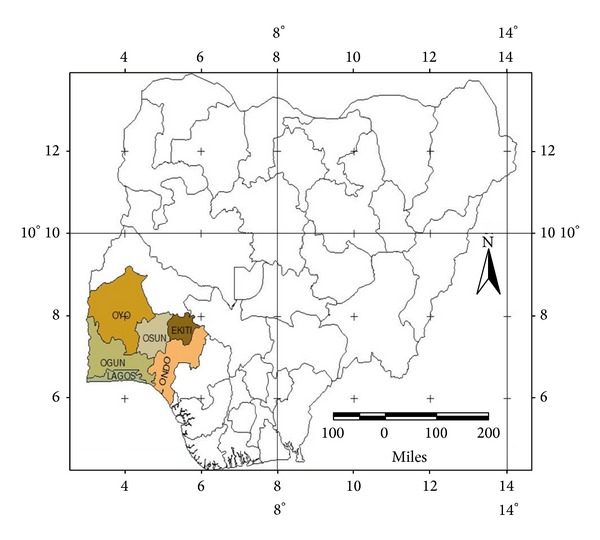
The Nigerian map showing the southwestern region.

The survey was carried out in three out of the six states that make up the southwest geopolitical zone of Nigeria (Figure 1).

### 2.2. Study Design

Sampling was purposive and the choice of rural hunting communities was based on accessibility, informed consent, and agreement to cooperate by dog owners. We sought the assistance of local government authority officials who are conversant with hunting communities in their areas and visits were paid to the communities to explain the nature of the research and what we required from the dog owners. Companion dog samples were obtained from dogs presented at veterinary hospitals with the cooperation of the owners and veterinary officers.

### 2.3. Sample Collection and Demographic Information

Samples were collected between December 2012 and April 2013. Blood (3 mL) was collected via the cephalic vein of each dog into plain glass bottles without anticoagulant; this was allowed to clot by sitting it undisturbed on the laboratory bench for 30 minutes (for samples collected from companion dogs in veterinary hospitals). Samples gotten on the field in rural communities were carefully stacked in slant position prior to being transported to the laboratory. All samples were then centrifuged at 1500 revolution per minute for 10 minutes and the separated sera were put in microcentrifuge tubes and stored at −20°C until needed [16, 17].

Demographic data collected during sampling include age (young: <1 year, adult: ≥1 year), sex (male or female), and location (rural or urban).

The owners were interviewed on their management practices as regards the use of dog and purpose of keeping dog (hunting or companion) and feeding. The reasons for some of the practices were also elucidated during the interview.

### 2.4. Serological Analysis

The prevalence of canine echinococcosis was determined using direct enzyme linked immunosorbent assay (ELISA) technique to detect* Echinococcus granulosus* antigen in dog serum. A commercial kit from Shenzhen Lvshiyuan Biotechnology Co, Ltd, China (Green spring canine echinococcosis ELISA antigen kit), was used and tests were carried out according to manufacturer's protocol.

### 2.5. Statistical Analysis

Statistical analysis was done using graph pad prism (version 5) with a *P* value of <0.05. Fisher's exact test was used to examine the relationship between sex, age, dog use (hunting or companion), and prevalence of canine echinococcosis.

## 3. Result

The study was carried out on 273 dogs (207 rural hunting and 66 urban companion dogs).

Most of the rural dwellers were superstitious and convincing them to have blood samples taken from their dogs was difficult. A good number that were approached refused to cooperate.

Total prevalence rate was 12.45% (34/273). Of the 207 hunting dogs sampled, 15.94% (33/207) was found to be positive while 1.52% (1/66) of companion dogs were positive showing a significant difference (*P* < 0.05). Of the 47 young dogs sampled, two were found to be positive, while 32 of the 226 adult dogs sampled were positive and there was no significant difference (*P* > 0.05) between the two groups. Between the sexes, no significant difference was observed (*P* > 0.05); with prevalence of 13.25% in female, 20 out of 151 samples were positive and with prevalence of 11.48% in male, 14 out of 122 samples were positive.

The owners of companion dogs that were interviewed confirmed that the dogs also doubled as guard dogs and are housed in cages or brick kernels within the confines of their fenced compounds. Only two of the owners claimed their dogs lived with them in the house. Feeding according to the owners ranged from cooked food and table scraps to compounded rations from personal formulas and available commercial dog foods. None admitted to feeding any form of raw food or meat. Most hunters however admitted feeding their hunting dogs with offal of the game caught while dressing. The belief expressed by majority is that the fresh blood is good for the dogs as carnivores and also sharpens their hunting skills. A few however claimed that they cook the offal before feeding the dogs with it as this in their opinion improves palatability. All the hunters allow their dogs to sleep outside their mud brick houses except nursing bitches and pups. We offered free treatment, which was rejected by the hunters; however antiectoparasite powders that were regarded as noninvasive were accepted.

## 4. Discussion

Reports on canine echinococcosis in Nigeria are scanty; however the studies on hydatidosis in farm animals [11, 18–22] have confirmed the presence of the infection. This means that the cycle is being completed in the final host though reports are few. The prevalence of 12.45% for canine echinococcosis obtained in this study is high for a country that has not paid adequate attention to the zoonotic infection. The use of serological methods for the diagnosis of canine echinococcosis has been recommended over the traditional arecoline purge [8], which has several limitations (cumbersome, dogs failing to purge, contraindication in pregnant bitches, aged dogs and young puppies, and sometimes death). The use of coproELISA technique for the diagnosis of canine echinococcosis serologically has been recommended [8] and applied successfully in several studies [23–26]. Its advantage over serum antibody detection is the high probability of correlation with current infection. The direct enzyme linked immunosorbent assay (ELISA) technique that we used to detect echinococcosis antigen in dog serum enjoys the advantage which coproELISA technique has over serum antibody detection.

The higher prevalence in hunting dogs could be attributed to the fact that they have limited access to veterinary care and hence lack adequate deworming. Most of the time, sample collection is compensated with provision of some routine treatment, basically the deworming of dogs sampled. However, most rural communities sampled were not very receptive to veterinary researchers and cooperation received was low. Most of the hunters refused the free veterinary care offered claiming that it would slow their dogs down from running properly and result in poor hunting performance. Further, hunting dogs have more access to infected carcass and the wild intermediate host in the bush [27], because apart from reports of infection in domestic ungulates, there have also been reports in wild ungulates, particularly bovids, as well as primates, leporids, and macropod marsupials [28, 29]. These dogs are also more likely to be fed raw viscera which might be infected due to the lack of knowledge of rural dwellers. This was confirmed from the hunters during sample collection, as they affirmed that viscera of the game caught by the dogs were fed to them uncooked as their prize.

Although it is assumed that there should be a lower worm burden in adult host compared to the younger host that has not yet acquired any immunity [30], the fact that more adult dogs were sampled when compared to the young dogs could be an explanation for the higher prevalence we obtained in adult dogs. Also, adult dogs are more predisposed to infection as they are likely to feed on raw infected carcass, are more active, and hence are more likely to be used for hunting, which heighten their risk of exposure to infection. Furthermore it is an established fact that immunosuppressed dogs exhibit more susceptibility to the infection [31] and rural dogs fall into this category as most of them are not properly fed and do not receive adequate medical attention. A higher number of females were sampled in this study probably due to the bias of most dog keepers in Nigeria for female dogs that are used for the purpose of dog breeding. Contrary to Budke's findings [32], which suggest that male canids are more likely to be infected with* Echinococcus* spp than females, the male dogs had a lower prevalence when compared to the female dogs and there is no relationship between sex and canine echinococcosis in this study.

## 5. Conclusion

The result of this study has brought to fore the presence of canine echinococcosis among dogs, especially in the rural communities. It also gives a strong indication of ongoing infection in the definitive hosts and this poses the risk of human cystic hydatid disease to exposed individuals. For a disease which the World Health Organization (WHO) has categorised as one of the most widespread parasitic diseases and also one of the most costly to be treated and prevented in terms of public health [8], the prevalence of 12.45% is of major concern not only because it is high but also because the country currently has no strategic control programme to prevent a serious public health situation.
